# Interprofessional Education as a Sandbox for Collaborative Play—Towards Health Equity

**DOI:** 10.1111/tct.70143

**Published:** 2025-06-27

**Authors:** Mark A. C. Versluis, Gail Jensen, Marco Antonio de Carvalho‐Filho, Steven J. Durning

**Affiliations:** ^1^ Department of Obstetrics and Gynaecology University Medical Center Groningen Groningen The Netherlands; ^2^ Wenckebach Institute (WIOO), Lifelong Learning, Education and Assessment Research Network (LEARN), University of Groningen University Medical Centre Groningen Groningen The Netherlands; ^3^ Center for Interprofessional Practice, Education and Research (CIPER), Creighton University Omaha Nebraska USA; ^4^ Department of Health Professions Education and Director, Center for Health Professions Education Uniformed Services University of the Health Sciences Bethesda USA

**Keywords:** equity, health professions education, healthcare, interprofessional education, under‐resourced setting

## 1 The ‘IPE‐Sandbox’

One of our first social experiences outside our families happens in primary school, playing in the sandbox. Conceptually, the primary school sandbox is a place where we play together under the watchful eyes of dedicated teachers. We interact, share, create, destroy, rebuild, fight and reconcile. We fail, stand up, move on and fail again. We negotiate and resolve conflict, and learn how to find common ground and a shared purpose. We learn to balance between taking the lead and giving space. We incorporate rules and internalize values. We learn about who we are and how we relate to others, growing as social beings who can respect and demand for respect. We feel proud of ourselves and celebrate when together, we achieve greatness.

The current sandbox of Interprofessional Education (IPE) falls short of this concept. IPE aims to provide students with the social experience and competencies to navigate a complex landscape of interprofessional practice [1, 2]. Sadly, Health Professions Education (HPE) is still shaped by a heritage of siloed professional education in many places in the world [3, 4, 5, 6]. It seems that most students in the IPE‐sandbox are building a sandcastle all by themselves and for themselves in parallel play, incapable of sharing their tools, ideas, challenges and achievements. There is limited interaction with peers from other professions and limited consideration of a shared objective in patient care. In this viewpoint, we reflect on our current IPE‐sandbox and explore how it could be optimized to offer more meaningful learning experiences by extending our gaze and learning from under‐resourced settings.

## 2 The Current Playing Field

The landscape of healthcare practice is more complex than ever as healthcare systems face challenges rooted in recent societal demands, such as improving health equity, combating structural racism and other forms of prejudice while creating an eco‐friendly future, and acknowledging a limited availability of resources [2, 7, 8, 9]. Increased life expectancy is accompanied by increased prevalence of chronic diseases and growing demand for diagnostic procedures and treatment options. Consequently, healthcare costs are spiraling out of control and an alarming shortage of health professionals threatens access to healthcare [2, 7, 9]. Inequity is particularly damaging when race, gender, socioeconomic status and other sociological aspects intersect to hamper health equity and health outcomes [7, 8, 9]. In this context of growing scarcity where health professionals already walk the extra mile, sharing the workload by improving interprofessional collaborative practice (IPCP) is essential to long‐term employability [2, 10]. Health equity and a sustainable workforce depend on learning from each other and crossing the borders of professional silos in a process of continuous development aiming to optimize care and resources [2, 5, 10, 11].

Responding to this need, many countries have engaged with the challenge of IPE implementation [4, 10]. North America has been successful with implementing IPE and developing competency frameworks that emphasize interprofessional values/ethics and patient centeredness among other interprofessional competencies [12, 13]. However, the majority of healthcare students worldwide graduate without any form of IPE [4, 14, 15, 16]. And even where implementation is successful, it is often unclear if IPE is successful in breaking professional siloes, improving IPCP and safeguarding health equity [3, 6]. There is a risk of implementing IPE while remaining stuck in parallel play, ticking the box for implementation but failing to result in improved IPCP [3, 4, 6, 15].

IPE that breaks professional silos, improves IPCP and safeguards health equity requires us to extend our networks beyond health professions to involve other professionals such as social scientists, engineers and policymakers [2]. To build healthcare networks that engage with patients and their ecology, addressing intersectionality in a holistic, meaningful way so that each person receives the care and attention they require. To prepare the next generation, we need to reconsider the kind of sand sculptures learners should build, when they should build them and with whom. Educators need to consider how students can best be guided in a socialization process of collaborative construction, looking beyond silos and parallel play, and seeing IPE as it could be—an innovative, evidence‐informed strategy that shapes health care for all.

## 3 Drawing a New ‘IPE‐Sandbox’

The new IPE‐sandbox should reflect the current landscape of practice and prepare for ever‐changing healthcare systems committed to social justice; supporting development of capability to collaborate with both health and non‐health professionals to address patients and their ecology; accepting diversity as an asset; and embracing the power of complementary expertise [6, 11]. This extended conceptualization of IPE should emphasize care for the sick as well as prevention, health promotion and meaningful connections with communities. Most healthcare systems, especially those in well‐resourced settings, have a traditional perspective on healthcare that revolves around hospitals caring for the sick, primarily a model of sick care instead of healthcare. However, to respond to the increase in healthcare demand, healthcare systems must shift the emphasis towards health promotion [2, 5, 9]. This shift in emphasis will also allow HPE to broaden the different contexts where IPE can be situated with the additional benefit that learners get a view of the intricate interplay between patients and their ecology in both hospital and community setting. The future IPE‐sandbox, therefore, is a place for evidence‐informed, context‐sensitive IPE activities where students in health and non‐health education learn by working together, co‐constructing knowledge and understanding of patient care, supported by meaningful social exchange in an collaborative effort to improve health for all people.

The new IPE‐sandbox should prepare for ever‐changing healthcare systems committed to social justice.

Educators can facilitate this by developing an interprofessional signature pedagogy and aligning with a future landscape of practice that extends beyond the medical domain [17]. The new IPE‐sandbox is a place where teachers support the learning process, acting as role models capable of creating a safe learning environment and breaking professional siloes. In many places, however, the teachers that oversee IPE are still of the same profession, with doctors teaching medical students and nurses teaching nursing students, both using the signature pedagogy characteristic of their profession [4, 15, 17]. As a result, students are presented with an outdated image of mono‐professional practice. To prepare students for interprofessional practice, the teacher community that oversees the sandbox should reflect the landscape of future practice, with teachers from different professions (health and non‐health) facilitating a reciprocal learning process, acting as role models and providing cross‐professional feedback, equipped with a signature pedagogy that embodies both professional and interprofessional values and beliefs [5, 17, 18].

In redesigning the IPE‐sandbox, we can learn from settings that are more regularly challenged by a care demand that does not align with available professionals such as warzones and/or the Global South. Under‐resourced healthcare systems often require flexibility and adaptability to break professional siloes. Pressured by lack of resources and personnel, care teams provide the best possible care, by shifting or delegating activities and responsibilities across professional boundaries, taking advantage of multi‐professional creativity. Illustrating the value for IPE, an ethnographic study investigating military interprofessional healthcare teams demonstrated the importance of teachers nurturing a growth mindset and a culture where *flailing is not failing* [19].

We can learn from settings that are challenged by a care demand that does not align with available professionals.

Extending our gaze to settings where care teams collaborate and function under the pressure of limited resources can be a source for frugal innovation for both IPC and IPE [20]. For example, the United Kingdom aims to adopt an interprofessional community‐based approach to primary care from Brazil [21]. Brazil's Family Health Strategy is characterized by primary care delivered by a range of healthcare professionals, including community health agents (i.e., appointed community members) that bridge the distance between their community and healthcare [22]. Embedded in their community, health agents are well positioned to provide outreach. Adopting such a program creates new opportunities for IPE to extend beyond the medical profession and promote health equity. Interestingly, community health centres in Brazil are collectively endowed with responsibility to educate as a team, facilitating role modeling of IPE teachers. Such a collective responsibility is not always obvious for health centres in well‐resourced settings.

Examples of healthcare interventions in the Global South further illustrate the possibilities to learn *from* and *in* these settings. For example, task shifting initiatives (i.e., initiatives where tasks are redistributed to maximize healthcare performance with an existing workforce), common to the Global South, provide a setting where boundary crossing is part of the daily routine [23]. Healthcare workers that are positioned fluidly across different professions can be teachers that embody interprofessionality. Supporting participants in task shifting projects to develop as teachers can provide a valuable addition to IPE faculty. An additional advantage of looking at healthcare systems in the Global South is that these systems commonly have a strong focus on community care and prevention, holding important lessons for healthcare systems that want to transition from sick care to healthcare [16]. Finally, the Global South encompasses different epistemologies that provide a valuable addition to established epistemologies from well‐resourced settings [14, 24, 25].

Healthcare workers that are positioned fluidly across different professions can be teachers that embody interprofessionality.

Making optimal use of under‐resourced settings to bolster IPE worldwide has implications for educators and researchers in IPE. Extending our gaze requires engaging in an open‐minded conversation about health and equity. Furthermore, under‐resourced settings need to become part of the academic conversation on IPE. There are plenty IPE‐initiatives in under‐resourced settings that offer unique opportunities to learn about implementation, faculty development and other aspects of IPE [14, 15, 16]. We call for a broader and more inclusive approach to IPE, in a reciprocal effort to further improve IPE and IPCP. To draw an improved IPE‐sandbox that extends beyond the medical domain, providing students with the social experience and competencies to navigate a complex landscape of interprofessional practice, engaging with patients in their ecology and guided by health equity as a core value.

Under‐resourced settings need to become part of the academic conversation on IPE.

## 4 Conclusion

IPE learners are often stuck in different sandboxes, in parallel play, by and for themselves. They are overseen by mono‐professional teachers, giving students a mono‐professional perspective of healthcare. We are missing out on the fun of the playful social interaction in the IPE‐sandbox where friction creates shine and students can grow as social beings and professionals that excel in interprofessional collaboration. We need to escape this situation by reconsidering the IPE‐sandbox, anticipating a future landscape of practice with more focus on health and health equity for our patients. We can do so by extending our gaze to include and learn from under‐resourced settings.

## Author Contributions

**Mark Versluis AC:** conceptualization, writing – original draft, writing – review and editing, investigation, resources. **Gail Jensen:** conceptualization, writing – review and editing, supervision. **Marco Antonio de Carvalho‐Filho:** conceptualization, writing – review and editing, supervision. **Steven Durning J:** conceptualization, writing – review and editing, supervision.

## Data Availability

Data sharing is not applicable to this article as no new data were created or analyzed in this study.
